# Personalized cancer care through biology informed treatment decisions

**DOI:** 10.1186/1878-5085-5-S1-A44

**Published:** 2014-02-11

**Authors:** Peter Kuhn

**Affiliations:** 1 Scripps Physics Oncology Center, USA

## 

Personalized cancer care depends substantially on three key elements i) modern interventions by surgery, radiation and therapeutics, ii) predictive diagnostics for each intervention, iii) predictive data models and mathematics that integrate each time point of each patient into larger populations. Patients with solid tumors have suffered particularly from the absence of routine access to relevant tumor tissue and precision diagnostics that predict response to therapy. Similarities can be drawn to the conversion of HIV from a deadly disease to a chronic condition through the ability to accurately understand the current viral load and prescribe the relevant drug cocktail. This same concept is now being followed in carcinomas by utilizing a simple blood sample as a fluid biopsy to extract individual cancer cells and conduct high-content single cell analysis for the development of predictive biomarkers.

The fluid phase of solid tumors is a clinical tool in personalized cancer care and an emerging research tool in basic science cancer discoveries. Utilizing the HD-CTC assay, we are undertaking a series of clinical studies investigating the metastatic pathways in cancer patients. The fluid phase of solid tumors is a critical third microenvironment in the development and progression of carcinomas. Cells originating from primary or secondary sites travel through the blood circulatory system to either get cleared out or initiate new tumor growth. Translational research efforts are attempting to identify the various subtypes of circulating tumor cells (CTCs), their origins, their destinations and their impact on the disease. Understanding and characterizing CTCs is a first step towards utilizing them as both biopsy material and directly as a biomarker. It requires approaches of subtyping CTCs at the single cell level using molecular and cellular approaches. The immediate use is the use of the HD-CTC assay as a fluid biopsy for therapeutic stratification.

The success of the fluid biopsy in real-time stratification of patient treatments will both improve patient outcomes by enabling higher treatment success rates and radically improve healthcare economics by reducing ineffective treatments.

